# Synthesis of CaF_2_ Nanoparticles Coated by SiO_2_ for Improved Al_2_O_3_/TiC Self-Lubricating Ceramic Composites

**DOI:** 10.3390/nano9111522

**Published:** 2019-10-25

**Authors:** Zhaoqiang Chen, Niansheng Guo, Lianggang Ji, Chonghai Xu

**Affiliations:** 1 School of Mechanical and Automotive Engineering, Qilu University of Technology (Shandong Academy of Sciences), Jinan 250353, China; G1758251323@163.com (N.G.); jilianggang123@126.com (L.J.); xch@qlu.edu.cn (C.X.); 2 Key Laboratory of Advanced Manufacturing and Measurement and Control Technology for Light Industry in Universities of Shandong, Qilu University of Technology (Shandong Academy of Sciences), Jinan 250353, China

**Keywords:** SiO_2_-coated CaF_2_, solid lubricant, self-lubricating ceramic tool, nanoparticles

## Abstract

In order to reduce the influence of CaF_2_ addition on the mechanical properties of self-lubricating ceramic tools, we applied a silicon dioxide (SiO_2_) coating on calcium fluoride (CaF_2_) nanoparticles through hydrolysis and condensation reactions using the tetraethoxysilane (TEOS) method. The powder was dried by the azeotropic method, so that it acquired a better dispersibility. The resulting composite powders were characterized using XRD (X-ray diffraction) and TEM (transmission electron microscopy), showing that the surface of CaF_2_ was coated with a layer of uniform and compact SiO_2_. SiO_2_ shells with different thicknesses could be obtained by changing the amount of TEOS added, and the thickness of the SiO_2_ shells could be controlled between 1.5 and 15 nm. At the same time, a ceramic material containing CaF_2_ nanoparticles and CaF_2_@SiO_2_-coated nanoparticles was prepared. It had the best mechanical properties when CaF_2_@SiO_2_-coated nanoparticles were added; its flexural strength, fracture toughness, and hardness were 562 ± 28 MPa, 5.51 ± 0.26 MPa·m^1/2^, and 15.26 ± 0.16 GPa, respectively. Compared with the ceramic tool containing CaF_2_ nanoparticles, these mechanical properties were increased by 17.57%, 12.67%, and 4.88%, respectively. The addition of CaF_2_@SiO_2_-coated nanoparticles greatly improved the antifriction and wear resistance of the ceramic material, and the antifriction and wear resistance were balanced.

## 1. Introduction

Calcium fluoride (CaF_2_) crystals have good optical properties, mechanical properties, and chemical stability; therefore, calcium fluoride is widely used [1,2,3,4,5]. Calcium fluoride crystals are very important optical functional crystals, which have the advantages of wide light transmission range, high transmittance, low refractive index, low dispersion, and so on and have become an irreplaceable lens material in ultraviolet lithography objective lens systems [6,7,8,9]. At the same time, calcium fluoride has low shear strength and thermophysical and thermochemical stability at high temperature, so it is used in high-temperature solid lubrication [10,11,12,13,14]. At present, calcium fluoride has been applied in the field of self-lubricating ceramics allowing made some advances [15,16,17,18,19,20]. Wu et al. [17] prepared an Al_2_O_3_/TiC/CaF_2_ multicomponent gradient self-lubricating ceramic composite by the hot-pressing method, showing that the addition of CaF_2_ could improve the antifriction properties of the ceramic composite. In the work by Kong et al. [21], a ZrO_2_–MoS_2_–CaF_2_ self-lubricating composite was prepared. A ZrO_2_ matrix composite showed a good tribological behavior over a wide temperature range with the addition of MoS_2_ and CaF_2_. On the one hand, the addition of calcium fluoride to self-lubricating ceramic materials will improve the wear resistance of the materials, while, on the other hand, it will reduce the mechanical properties of the materials and the overall reliability of the ceramic tool. One of the main problems to be solved is how to maintain high mechanical properties of self-lubricating ceramic tools together with high lubricating performance.

With the development of nanotechnology, the properties of nanomaterials, such as small size effect and macroscopic quantum-tunneling effect, have been attracted more and more attention. In particular, preparation and applications of nano-calcium fluoride have been greatly developed [22,23,24,25]. The introduction of nano-materials can improve the mechanical properties as well as the friction and wear properties of composite ceramic materials [26,27,28,29,30]. At the same time, because nanoparticles have high surface energy and chemical activity, they can be easily deposited on a worn surface during the friction process, forming a protective layer with low melting point and easy shearing and thus playing a good anti-friction and anti-wear role [31,32,33,34]. However, nano-materials have the problems of small particle size, large specific surface area, and high surface activity, which make nano-materials easy to agglomerate, thus affecting their performance [35,36]. Powder coating refers to the process of adsorbing or coating another substance or substances on the surface of a powder to form a composite material with a core–shell structure. Powder coating can change the physical and chemical properties of a powder [37,38,39,40,41,42,43,44]. Hu et al. [41] successfully coated SiO_2_ on the surface of monodisperse CoFe_2_O_4_, proving that the silica coating can prevent the aggregation and growth of nanoparticles at high temperature, making the nanoparticles suitable for various high-temperature technological applications. Wu et al. [42] prepared h-BN@Ni powders, which, compared to h-BN powders, greatly improved the mechanical properties of self-lubricating tools. In the work by Zhang et al. [43], a core–shell nanocomposite with polytetrafluoroethylene as the core and polymethyl methacrylate as the shell was prepared. The mechanical and lubricating properties of the nanocomposite were significantly improved.

In self-lubricating ceramic tools, the direct addition of calcium fluoride will significantly reduce the mechanical properties of the cutting tools, because the mechanical properties of calcium fluoride are relatively low [17,19]. Therefore, core–shell coating of calcium fluoride has been used to maintain simultaneously high mechanical properties and lubricity of the cutting tools [45]. In this paper, a layer of silicon dioxide was successfully coated on the surface of CaF_2_ nanoparticles through hydrolysis and condensation reactions with the tetraethoxysilane (TEOS) method. The powder coating was combined with nano powder, and the CaF_2_@SiO_2_-coated nanoparticles were added to replace CaF_2_ nanoparticles in the self-lubricating ceramic tool. The mechanical properties and the wear resistance of the ceramic tool were greatly improved, and the ceramic tool had antifriction properties.

## 2. Experimental Procedure

### 2.1. Materials and Processing

The starting materials used to prepare the CaF_2_@SiO_2-_coated nanoparticles are commercially available: Ca(NO_3_)_2_ (purity > 99.9%, Shanghai Fine Chemical Co., Ltd., Shanghai, China), NH_4_F (analytically pure, Tianjin Chemical Reagent Factory, Tianjin, China), NH_3_H_2_O (analytically pure, Tianjin Chemical Reagent Factory, Tianjin, China), TEOS analytical reagent (Tianjin Botong Chemical Co., Ltd., Tianjin, China), n-butanol, distilled water, and absolute ethanol were used as received without further purification.

### 2.2. Synthesis of CaF_2_@SiO_2_ Powders

According to the molar ratio of 1:1.5, calcium nitrate and ammonium fluoride were weighed and dissolved in equal volumes of absolute ethyl alcohol and water, respectively, and stirred until completely dissolved; then, an absolute ethyl alcohol solution containing polyethylene glycol (PEG) was added, and ultrasonic dispersion and mechanical stirring were carried out for 20 min to uniformly disperse the calcium nitrate and ammonium fluoride. A calcium nitrate dispersion was slowly added into the ammonium fluoride dispersion through a constant-pressure separatory funnel, and ultrasonic stirring was continuously carried out during the process. After reacting for 30 min, the mixture was left standing for 2 h. The obtained product was centrifuged for 30 min at 4000 r/min, washed with distilled water for 3 times, and azeotropically dried to obtain a nano-calcium fluoride powder. Then, 1 g of self-made nano-CaF_2_ powder was weighed, and 100 mL of absolute ethyl alcohol solution and a proper amount of dispersant polyvinylpyrrolidone (PVP) were added, followed by ultrasonic dispersion for 40 min and heating in a water bath under rapid stirring, while keeping the temperature between 35 and 45 °C. Distilled water (2.5 mL) was added to the above solution, and the pH was adjusted to 8.5 by adding an appropriate amount of ammonia water. To the above mixed solution, 1–4 mL of TEOS was slowly added dropwise, after which the mixture was continuously heated and rapidly stirred for 1 h. The obtained suspension was centrifuged at 6000 r/min for 25 min, then washed with anhydrous ethanol for 3 times. After cleaning, a wet gel was added into a 6:4 solution of n-butanol and distilled water, and after ultrasonic stirring for 30 min, the powder was azeotropically dried to obtain the CaF_2_@SiO_2_ composite powder with nano-CaF_2_ as core and SiO_2_ as shell.

### 2.3. Preparation of the Self-Lubricating Ceramic Tool Materials

The purity of each raw particle was higher than 99.9%. The average particle size of each particle were as follows: Al_2_O_3_ powder, 0.5 µm, TiC, 0.5 µm, MgO, 1 µm; the average size of the core–shell solid lubricant composite particles CaF_2_ made in our own laboratory was 30–50 nm.

The Al_2_O_3_/TiC/CaF_2_@SiO_2_ (ATCS) self-lubricating ceramic composite was prepared by the vacuum hot-pressing sintering technique. The sintering temperature was 1650 °C, the heating rate was 20 °C/min, the holding time was 20 min, and the hot-pressing pressure was 30 MPa. The volume ratio of commercially available, high-purity Al_2_O_3_ to TiC was 7:3, while the volume fraction of CaF_2_@SiO_2_ was 10%. For comparison, the Al_2_O_3_/TiC/CaF_2_(ATC) self-lubricating ceramic composite was prepared under the same conditions.

### 2.4. Performance Testing of Tool Materials

The resulting ceramic embryo body obtained was processed into a standard sample with a size of 3 mm × 4 mm × 30 mm to test the mechanical properties of the material. A bending strength test was performed by a three-point bending method with a span of 20 mm and a displacement loading speed of 0.5 mm/min. Vickers hardness was measured by a Hv-120 Vickers hardness tester with an indentation load of 196 N and dwell time of 15 s. Fracture toughness was measured by the indentation method, and fracture toughness was determined by indentation crack length [42].

### 2.5. Characterization

X-ray diffraction (XRD, D8-ADVANCE, Bruker AXS Co., Karlsruhe, Germany) was used for phase identification of the CaF_2_ nanoparticles and CaF_2_@SiO_2_-coated nanoparticles. X-ray diffraction was carried out using Cu Kα radiation with 40 kV and 40 mA, and the samples were analyzed at room temperature over a 2θ range from 15° to 80° and at a scanning rate of 10°/min. Morphology and crystallinity of the CaF_2_ nanoparticles and CaF_2_@SiO_2_-coated nanoparticles were obtained by transmission electron microscopy (TEM, JEM-1400, JEOL, Tokyo, Japan). The fracture morphology of the ceramic tools was examined and analyzed using a field-emission scanning electron microscope (SEM, Regulus8220, HITACHI, Tokyo, Japan), along with an energy-dispersive spectroscope (EDS).

## 3. Results and Discussion

### 3.1. Characterization of Structure and Morphology

**Figure 1:**
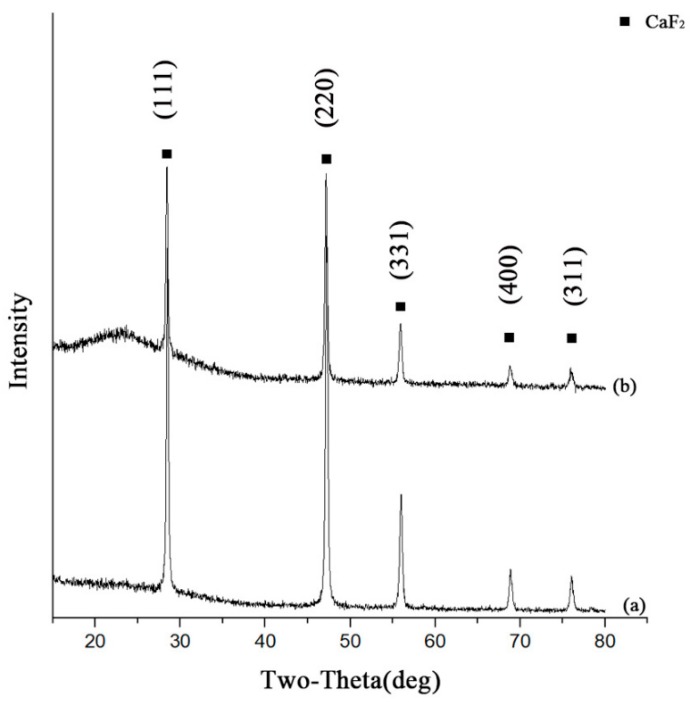
XRD patterns of (**a**) pure CaF_2_ and (**b**) CaF_2_@SiO_2_.

Figure 1 shows the XRD patterns of the as-prepared CaF_2_@SiO_2_ nanoparticles and pure CaF_2_ nanoparticles. Figure 1a shows the XRD patterns of CaF_2_ nanoparticles. All the discernible peaks were in good agreement with the data of pure cubic CaF_2_ crystals (JCPDS NO.35–0816). The diffraction peak of CaF_2_ crystals was narrow and sharp, which indicated that the prepared CaF_2_ had high crystallinity, and no impurity peak was detected, indicating that the prepared CaF_2_ had high purity. As shown in Figure 1b, the XRD patterns of CaF_2_@SiO_2_ nanoparticles were the same as the XRD patterns of pure cubic CaF_2_ crystals; only at about 2θ = 20°–25°, the pattern for CaF_2_@SiO_2_ had a low and wide peak, attributed to a silica dioxide amorphous halo [37,41].

**Figure 2:**
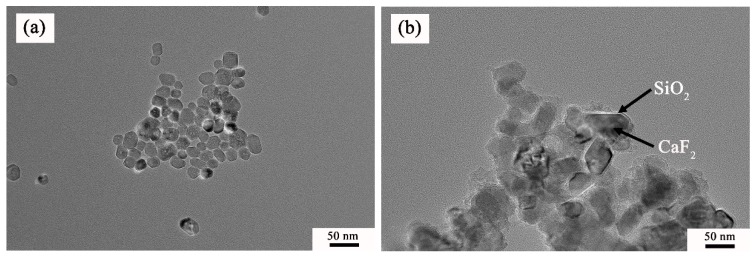
TEM micrographs of (**a**) CaF_2_ and (**b**) CaF_2_@SiO_2_.

**Figure 3:**
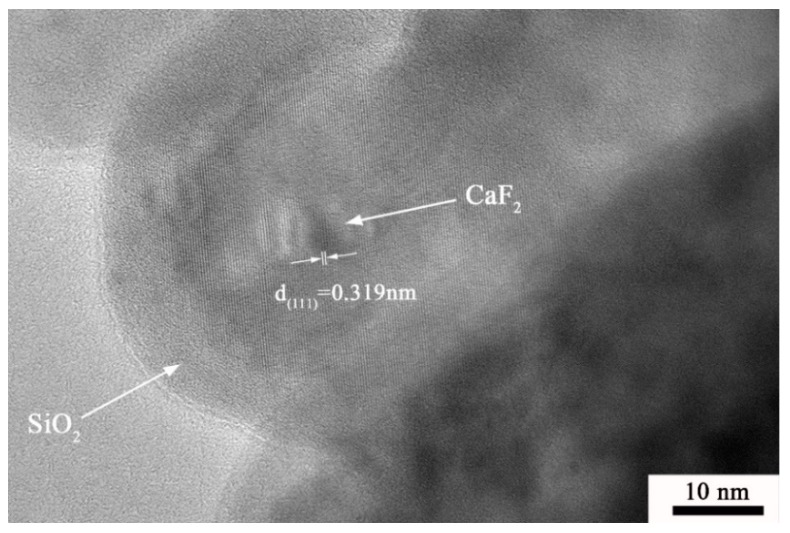
HRTEM micrographs of CaF_2_@SiO_2_.

The TEM images of the pure CaF_2_ and CaF_2_@SiO_2_ nanoparticles are shown in Figure 2. Figure 2a shows a transmission electron microscope image of pure CaF_2_. As shown in Figure 2a, the CaF_2_ nanoparticles had good dispersibility and an approximately round flake structure, and the average particle size of nano CaF_2_ was about 30–50 nm. Figure 2b shows a transmission electron microscope image of CaF_2_@SiO_2_ nanoparticles. The TEM micrograph clearly shows that the surface of the CaF_2_ nanoparticles was covered by a layer of amorphous SiO_2_, and the smooth edge of the CaF_2_ was tightly covered by amorphous SiO_2_. The coated powder had good dispersibility, and the average thickness of the amorphous silica coating was about 3.6 nm. The thickness of the SiO_2_ shell in the coated CaF_2_@SiO_2_ nanoparticles can be regulated and controlled; it can be changed by varying the amount of TEOS added. Figure 3 is a high-resolution transmission electron microscopy (HRTEM) image of CaF_2_@SiO_2_-coated nanoparticles. The HRTEM image shows that the lattice fringes were about 0.319 nm, which corresponds to the (111) orientation of CaF_2_. Amorphous SiO_2_ was evenly coated on the edge of calcium fluoride, and SiO_2_ and the CaF_2_ tightly combined. This shows that SiO_2_ was successfully coated on the surface of CaF_2_ nanoparticles, providing a uniform coating and a good coating effect.

### 3.2. Effect of TESO Addition on Coating Thickness

**Figure 4:**
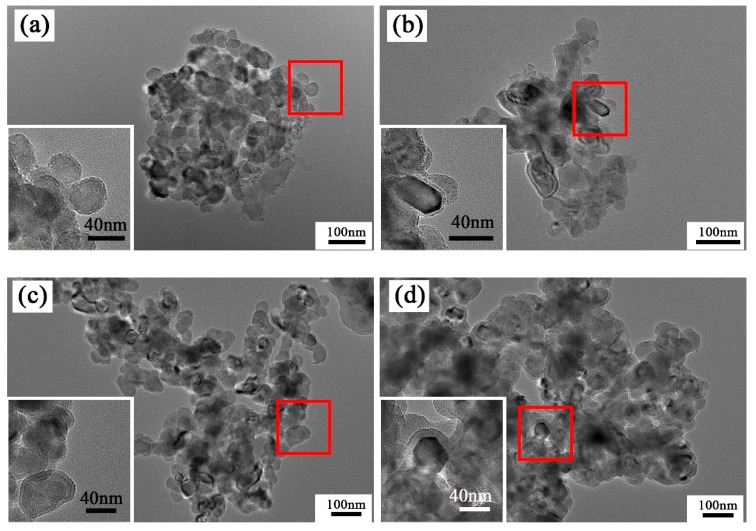
TEM micrographs in the presence of different tetraethoxysilane (TEOS) amounts. (**a**) 1 mL (**b**) 2 mL (**c**) 3 mL (**d**) 4 mL of TEOS.

TEM images of CaF_2_@SiO_2_-coated powder with different amounts of TEOS are shown in Figure 4. The amount of CaF_2_ was 1 g, the pH value was 8.5, and the amounts of TEOS were 1 mL, 2 mL, 3 mL, and 4 mL. The white framed images are partial enlarged views of the red selected areas. Figure 4a shows the powder obtained when 1 mL of TEOS was added; it can be seen from the figure that this TEOS amount resulted in less amorphous SiO_2_. At the edge of nano CaF_2_, the thickness of the SiO_2_ shell was very small, about 1.2 nm. When the amount of TEOS added was increased to 2 mL (Figure 4b), the thickness of the SiO_2_ shell coated on the nano-CaF_2_ increased to about 3.6 nm, but the coating effect was poor, and the thickness of the SiO_2_ shell was uneven. As shown in Figure 4c, when the amount of TEOS added was 3 mL, the thickness of the SiO_2_ shell increased to about 5.8 nm. At the same time, it can be seen that the amorphous SiO_2_ coated on CaF_2_ had uniform thickness and a good coating effect, but slight agglomeration occurred. When the amount of TEOS further increased to 4 mL, the thickness of the SiO_2_ shell reached about 13.3 nm, the coating thickness was relatively uniform, but agglomeration was more pronounced. The thickness of the SiO_2_ shell coated on CaF_2_ increased with the increase of the amount TEOS, but when the amount of TEOS added was too large, agglomeration occurred, whereas when the amount of TEOS was 3 mL, the best coating effect was obtained. SiO_2_ shells with different thicknesses could be obtained by changing the amount of TEOS added, and the thickness of the SiO_2_ shell could be controlled between 1.5 and 15 nm.

### 3.3. Improvement in Mechanical Properties and Microstructurs

**Figure 5:**
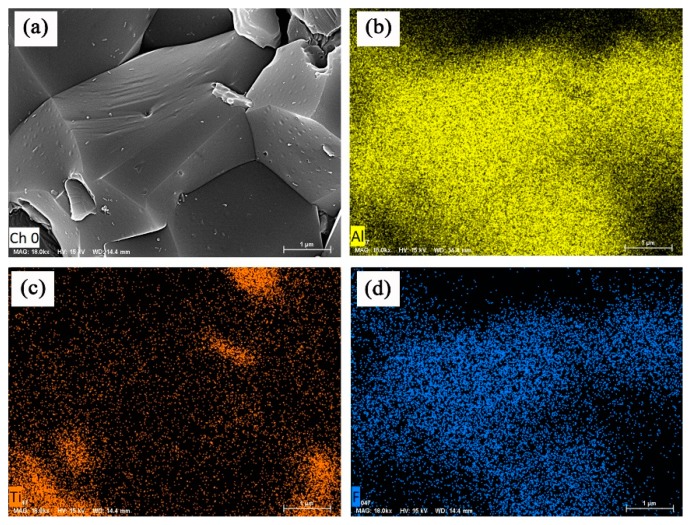
SEM micrograph and EDS spectra of fracture surfaces of Al_2_O_3_/TiC/CaF_2_ self-lubricating ceramic composites: (**a**) fracture morphology of Al_2_O_3_/TiC/CaF_2_ ceramic composites (**b**) Al element (**c**) Ti element (**d**) F element.

The scanning electron micrographs and EDS spectra of the fracture surfaces of the Al_2_O_3_/TiC/CaF_2_ ceramic composite are shown in Figure 5. EDS analysis results showed that the larger crystal grains in the Figure 5 were alumina crystals. According to the distribution of the F element, it can be seen that a large amount of uniformly dispersed nano-calcium fluoride was distributed on the surface of the alumina crystal grains. From the SEM images of the fracture surfaces, it can be seen that fine white protrusions were uniformly distributed on the surface of the alumina grains. These white protrusions were self-made nano-calcium fluoride grains, which formed an in-crystal nanostructure. The fracture mode of the ceramic composites was mainly intergranular fracture, with a small amount of transgranular fracture.

**Figure 6:**
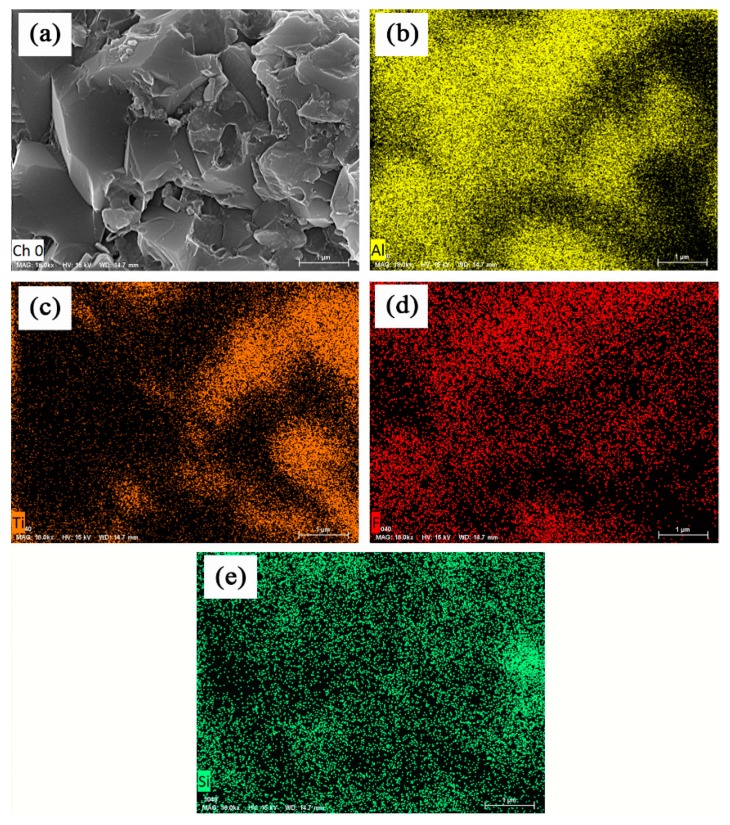
SEM micrograph and EDS spectra of fracture surfaces of Al_2_O_3_/TiC/CaF_2_@SiO_2_ self-lubricating ceramic composites: (**a**) fracture morphology of Al_2_O_3_/TiC/CaF_2_@SiO_2_ ceramic composites (**b**) Al element (**c**) Ti element (**d**) F element (**e**) Si element.

The scanning electron micrographs and EDS spectra of the fracture surfaces of the Al_2_O_3_/TiC/CaF_2_@SiO_2_ ceramic composite are shown in Figure 6. EDS analysis results showed that Si elements were mostly distributed at the grain boundaries between the right-hand grains. From the SEM images of the fracture surfaces, it can be seen that the nano-CaF_2_ protrusions on the surface of the alumina grains decreased, while the alumina grains became fine and the grain boundaries between the grains became blurred. Transgranular fracture increased, and intergranular fracture occurred in the ceramic composite. The fracture mode of the tools was mainly transgranular fracture. Transgranular fracture consumes a large amount of fracture energy, which is helpful to improve the mechanical properties of tool materials, making them compact with less defects. The addition of the coating powder plays a role in refining the crystal grains of a ceramic composite, improving the mechanical properties of the ceramic composite, enhancing the interfacial bonding force of the ceramic matrix material, changing the main fracture mode of the cutter, and improving the mechanical properties of the ceramic composite.

Table 1 shows the mechanical properties of ceramic tools with CaF_2_ nanoparticles and CaF_2_@SiO_2_-coated nanoparticles. Compared with the ceramic tool with CaF_2_ nanoparticles, the ceramic tool with CaF_2_@SiO_2_-coated nanoparticles had a hardness increase of 4.88%, a flexural strength increase of 17.57%, a fracture toughness increase of 12.67%. The flexural strength of the ceramic tool was also greatly improved, which was due to the change of the main fracture mode in the ceramic tool material; the hardness and fracture toughness of the ceramic tool were also improved. The addition of CaF_2_@SiO_2_-coated nanoparticles greatly improved the antifriction and wear resistance of the ceramic tool material; the antifriction and wear resistance of the tool material were balanced.

## 4. Conclusions

In this paper, SiO_2_ was successfully coated on the surface of CaF_2_ nanoparticles to prepare a nano-powder with a core–shell structure. After adding CaF_2_ nanoparticles and CaF_2_@SiO_2_-coated nanoparticles into an Al_2_O_3_/TiC ceramic matrix, the mechanical properties and the micro-morphology of ceramic tools were analyzed. The effects of adding CaF_2_@SiO_2_-coated nanoparticles on the micro-morphology and mechanical properties of the ceramic tools were compared with those of adding CaF_2_ nanoparticles. The following conclusions were obtained.

1. SiO_2_ shells with different thicknesses could be obtained by changing the amount of TEOS added, and the thickness of the SiO_2_ shell could be controlled between 1.5 and 15 nm. However, when the TEOS amount was too large, agglomeration occurred, whereas when the TEOS amount was 3 mL, the best coating effect was obtained.
2. The ceramic tool had the best mechanical properties when CaF_2_@SiO_2_-coated nanoparticles were added. The flexural strength, the fracture toughness, and the hardness were 562 ± 28 MPa, 5.51 ± 0.26 MPa·m^1/2^ and 15.26 ± 0.16 GPa, respectively. Compared with the ceramic tool with the CaF_2_ nanoparticles, the above performances were increased by 17.57%, 12.67% and 4.88%, respectively.
3. Compared with the ceramic tool with CaF_2_ nanoparticles, the ceramic tool with CaF_2_@SiO_2_-coated nanoparticles showed a great change in its microscopic morphology. The addition of the coated powder played a role in refining the crystal grains of the ceramic tool and, at the same time, increased its transgranular fracture, improving its performance.

## Figures and Tables

**Table 1:** Mechanical properties of the ceramic tool material.

Material	Flexural Strength/MPa	Fracture Toughness/MPa·m^1/2^	Hardness/GPa
ATC	478 ± 21	4.89 ± 0.13	14.55 ± 0.19
ATCS	562 ± 23	5.51 ± 0.21	15.26 ± 0.16
